# Supplementary figures and images for: Inhibition of the Notch signal transducer CSL by Pkc53E-mediated phosphorylation to fend off parasitic immune challenge in Drosophila
Source: eLife. 2024 Nov 6;12:RP89582. doi: 10.7554/eLife.89582 (PMC11540305; doi:10.7554/eLife.89582)

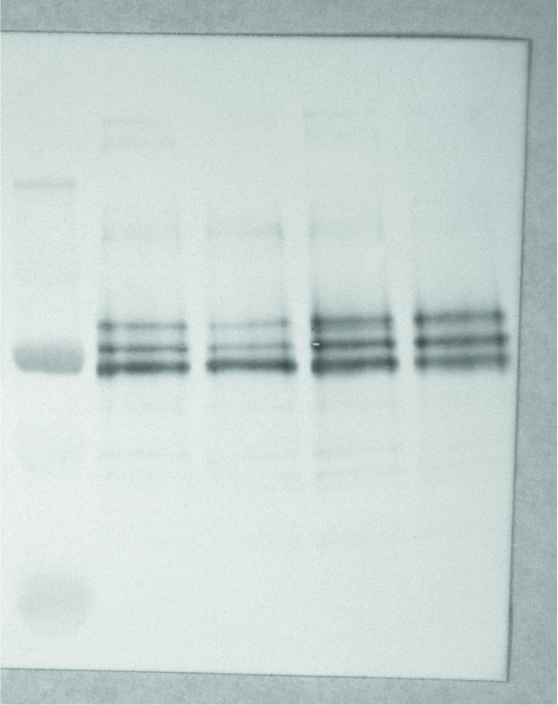

Supplement: Figure 2—source data 1. [file elife-89582-fig2-data1.zip › Figure 2B anti-mCh.jpg]

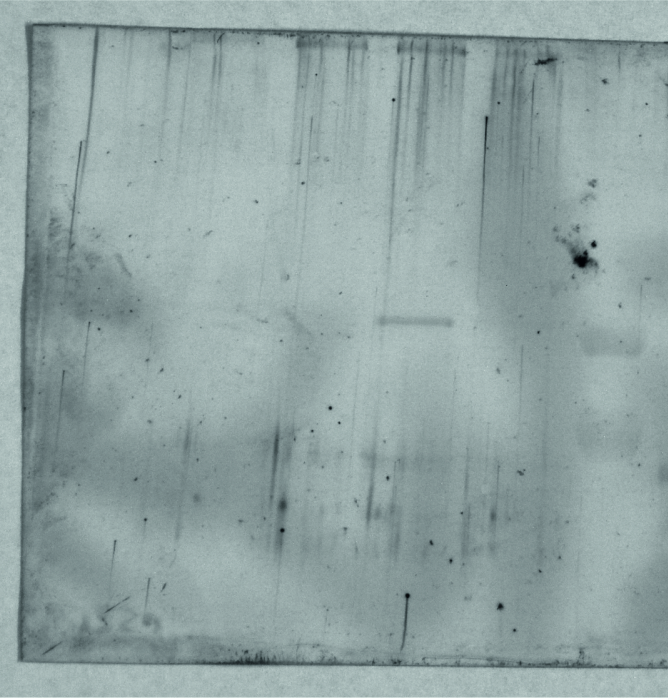

Supplement: Figure 2—source data 1. [file elife-89582-fig2-data1.zip › Figure 2B anti-pS269.jpg]

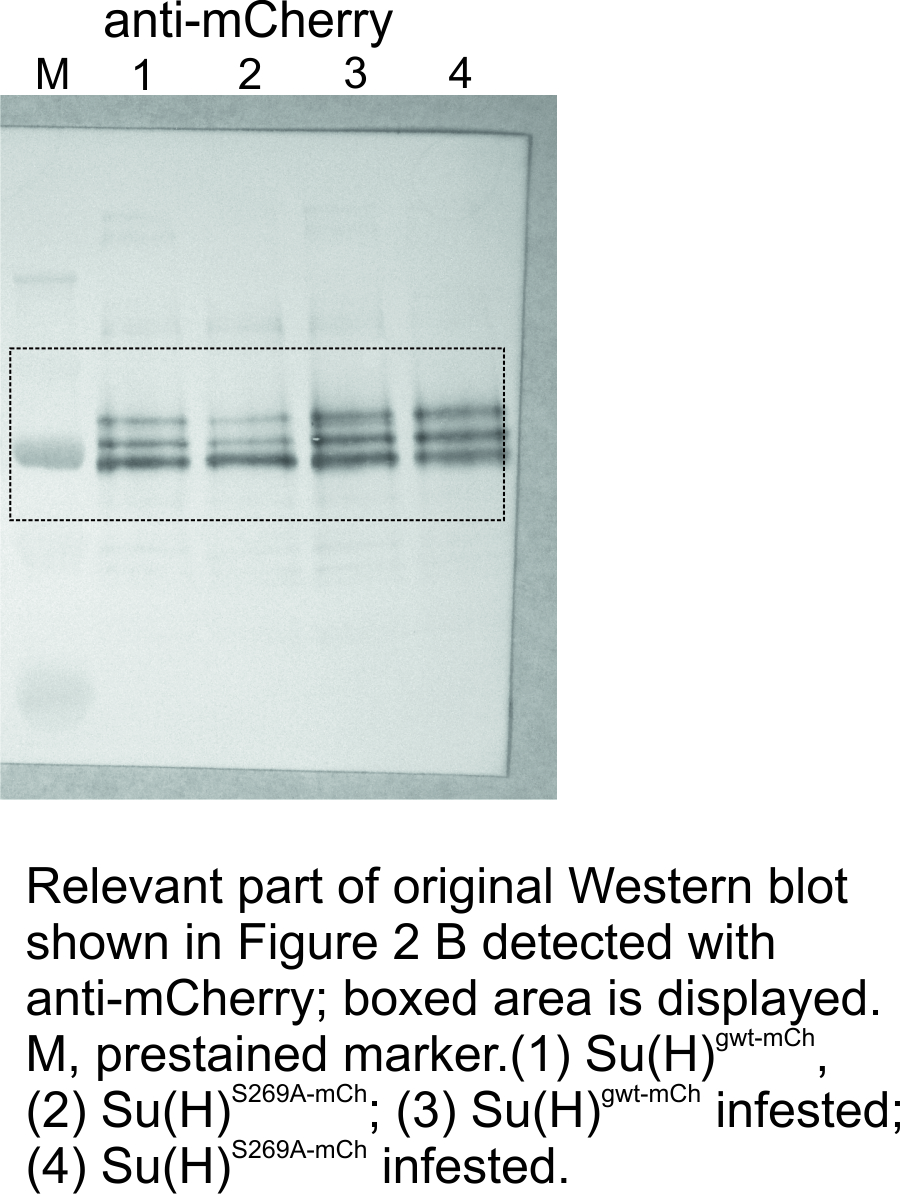

Supplement: Figure 2—source data 2. — Boxed areas correspond to regions shown in the main figure. [file elife-89582-fig2-data2.zip › Figure 2B anti-mCh, labelled.jpg]

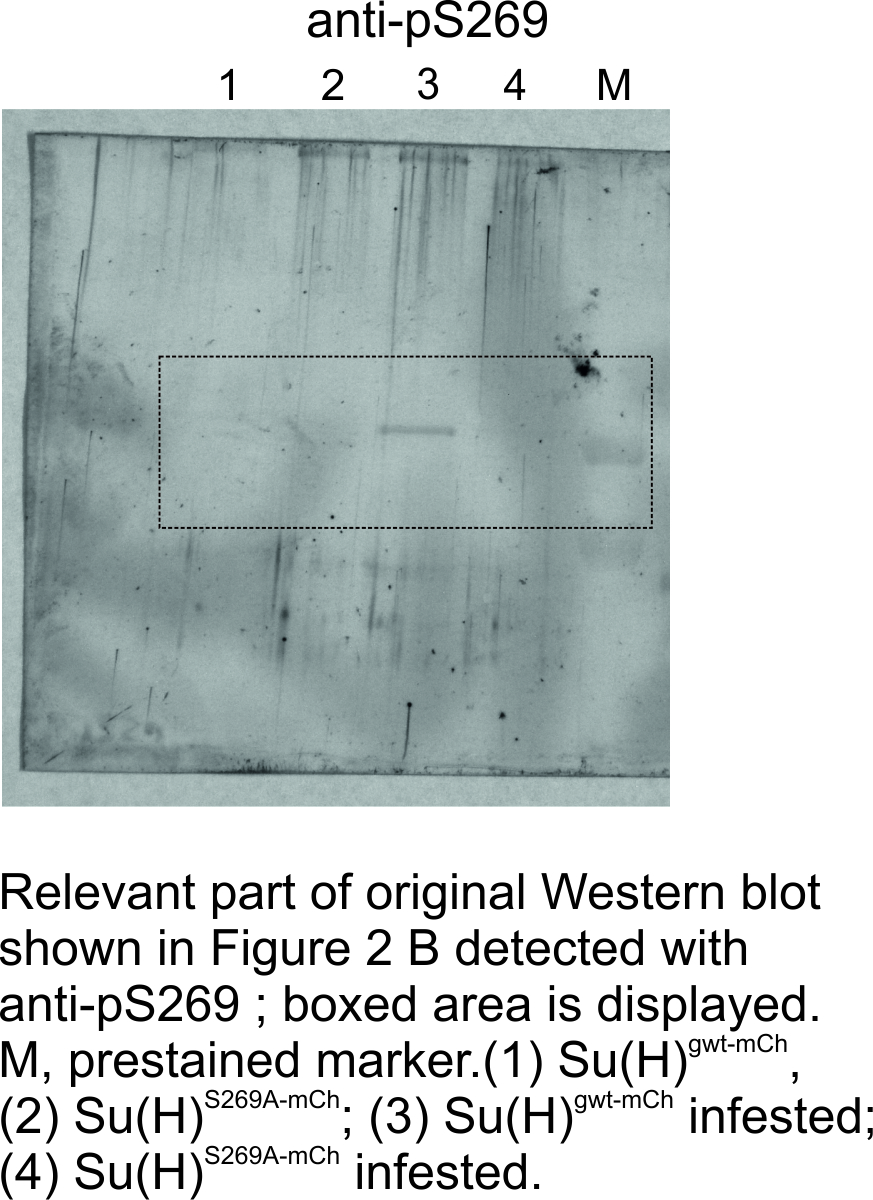

Supplement: Figure 2—source data 2. — Boxed areas correspond to regions shown in the main figure. [file elife-89582-fig2-data2.zip › Figure 2B anti-pS269, labelled.jpg]

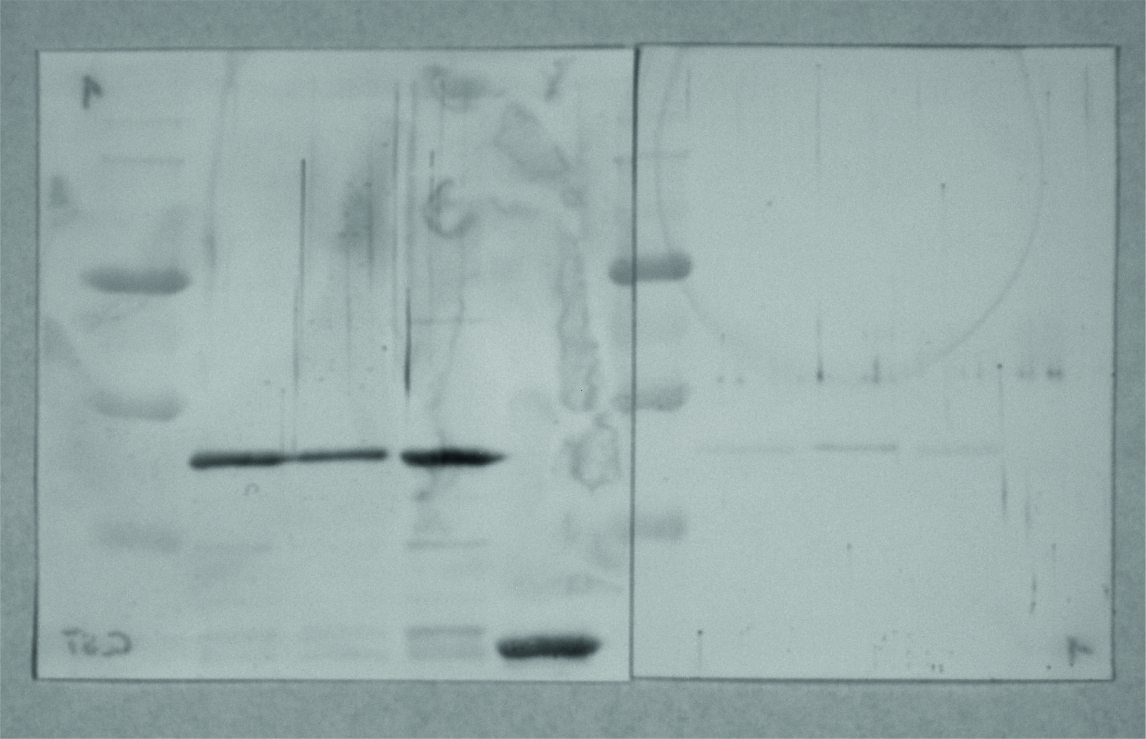

Supplement: Figure 2—figure supplement 1—source data 1. [file elife-89582-fig2-figsupp1-data1.zip › Figure 2-Figure Supplement 1-Source Data 1.jpg]

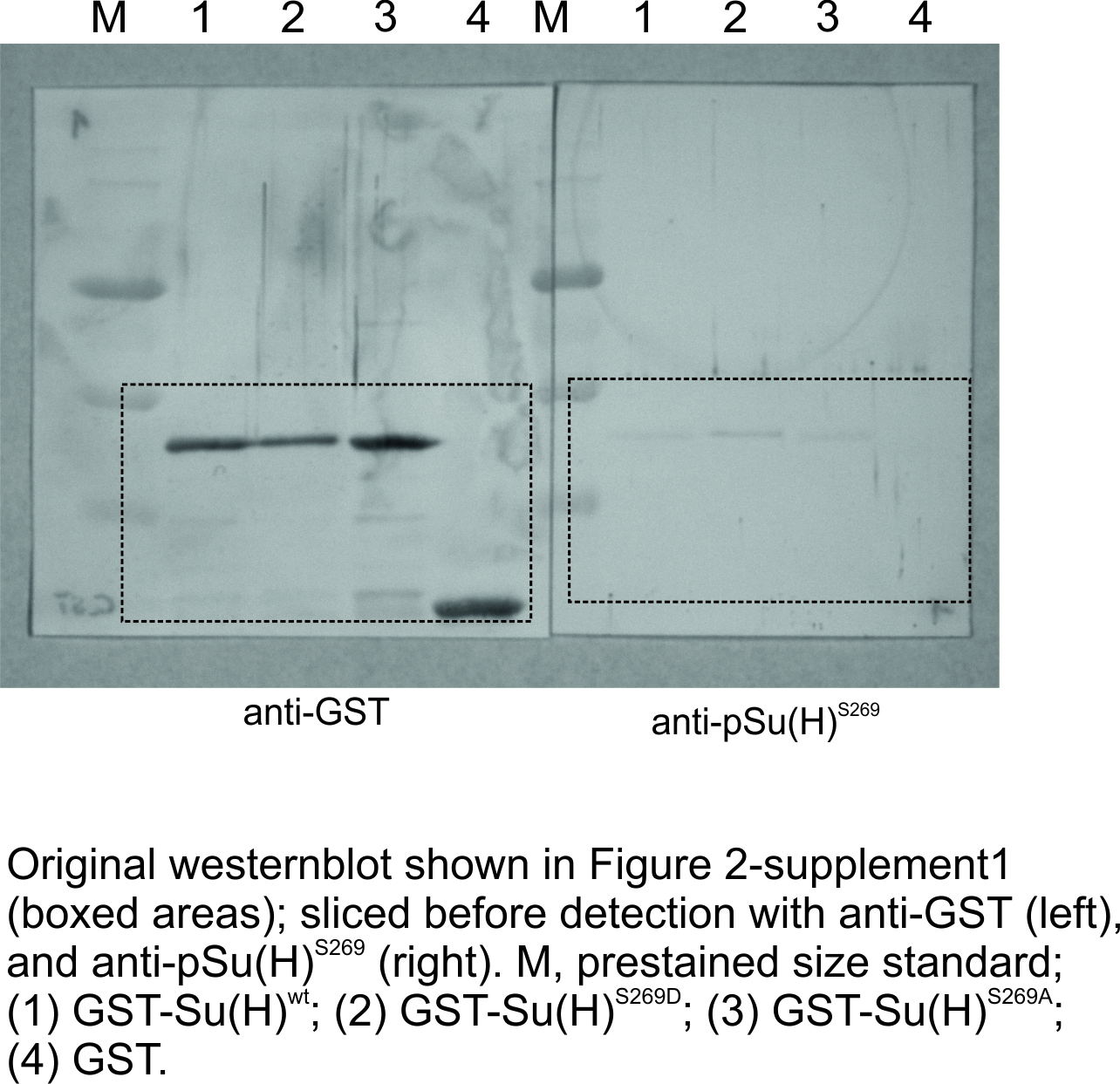

Supplement: Figure 2—figure supplement 1—source data 2. — Boxed areas correspond to regions shown in the main figure. [file elife-89582-fig2-figsupp1-data2.zip › Figure 2-Figure Supplement 1-Source Data, labelled.jpg]

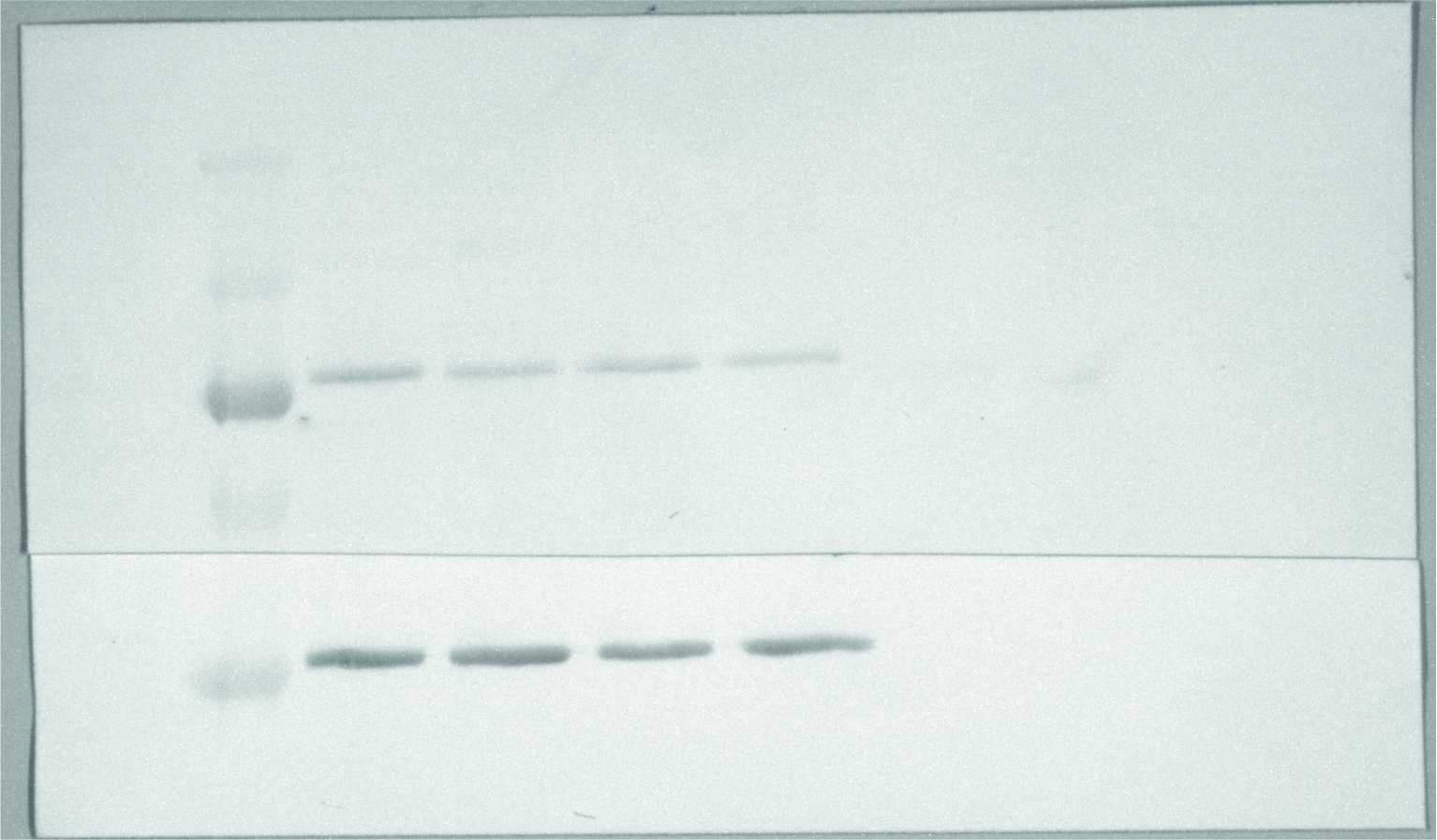

Supplement: Figure 5—figure supplement 1—source data 1. [file elife-89582-fig5-figsupp1-data1.zip › Figure 5-Figure Supplement 1-Source Data 1.jpg]

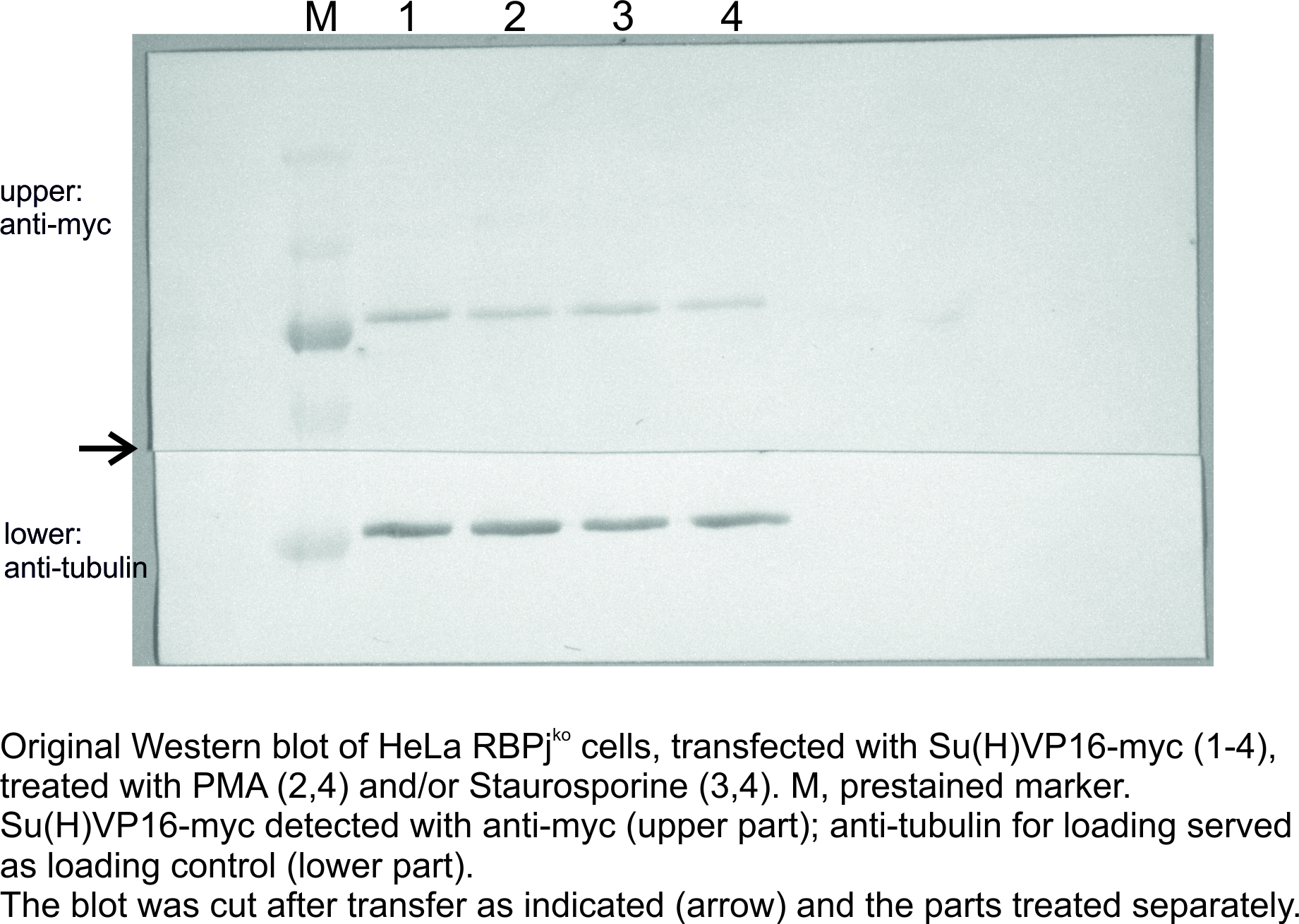

Supplement: Figure 5—figure supplement 1—source data 2. [file elife-89582-fig5-figsupp1-data2.zip › Figure 5-Figure Supplement 1-Source Data, labelled.jpg]

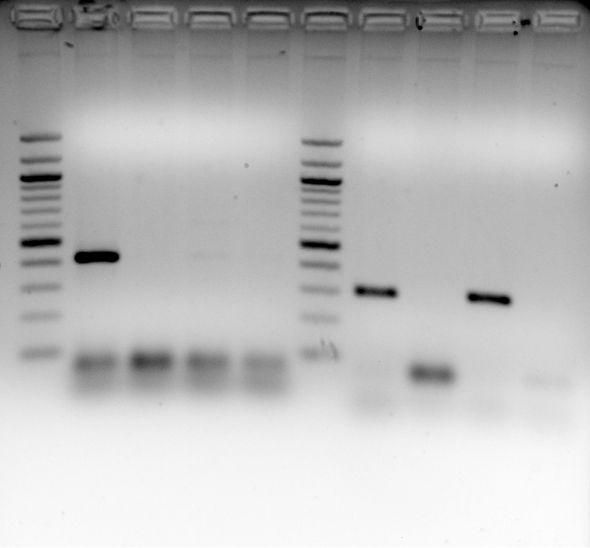

Supplement: Figure 6—figure supplement 1—source data 1. [file elife-89582-fig6-figsupp1-data1.zip › Figure 6-Figure Supplement 1-Source Data 1.jpg]

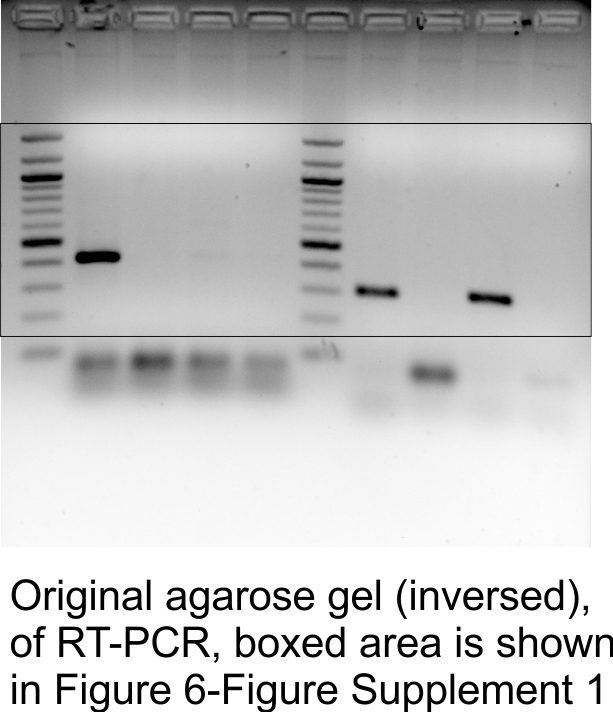

Supplement: Figure 6—figure supplement 1—source data 2. — Boxed area corresponds to region shown in the main figure. [file elife-89582-fig6-figsupp1-data2.zip › Figure 6-Figure Supplement 1-Source Data, labelled.jpg]

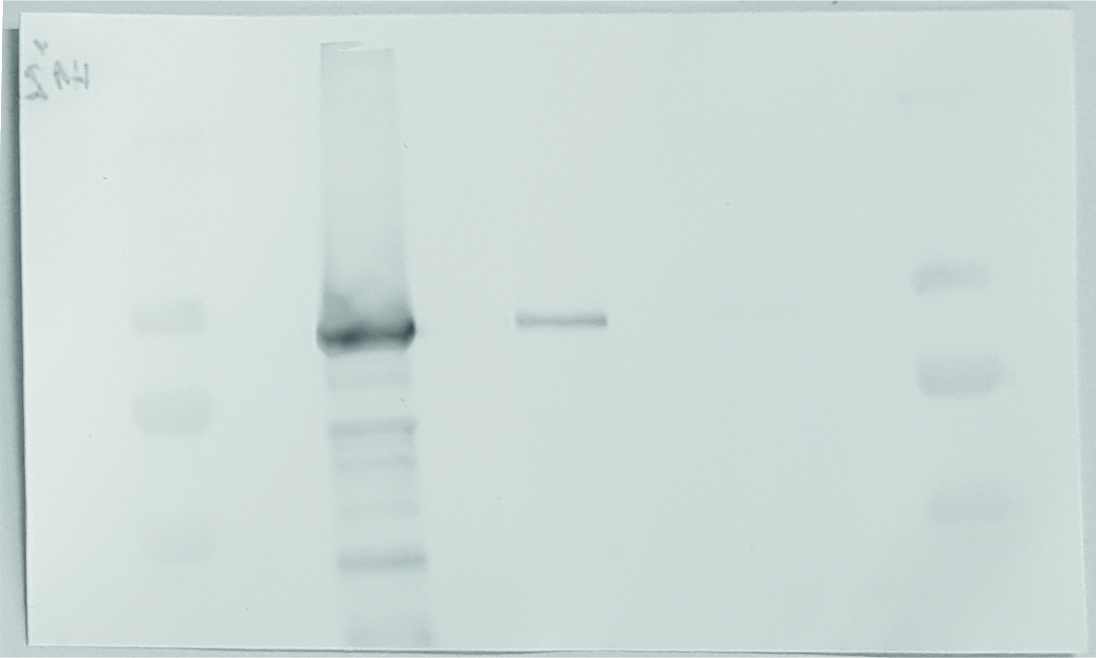

Supplement: Figure 7—source data 1. [file elife-89582-fig7-data1.zip › head co-IP anti-HA.jpg]

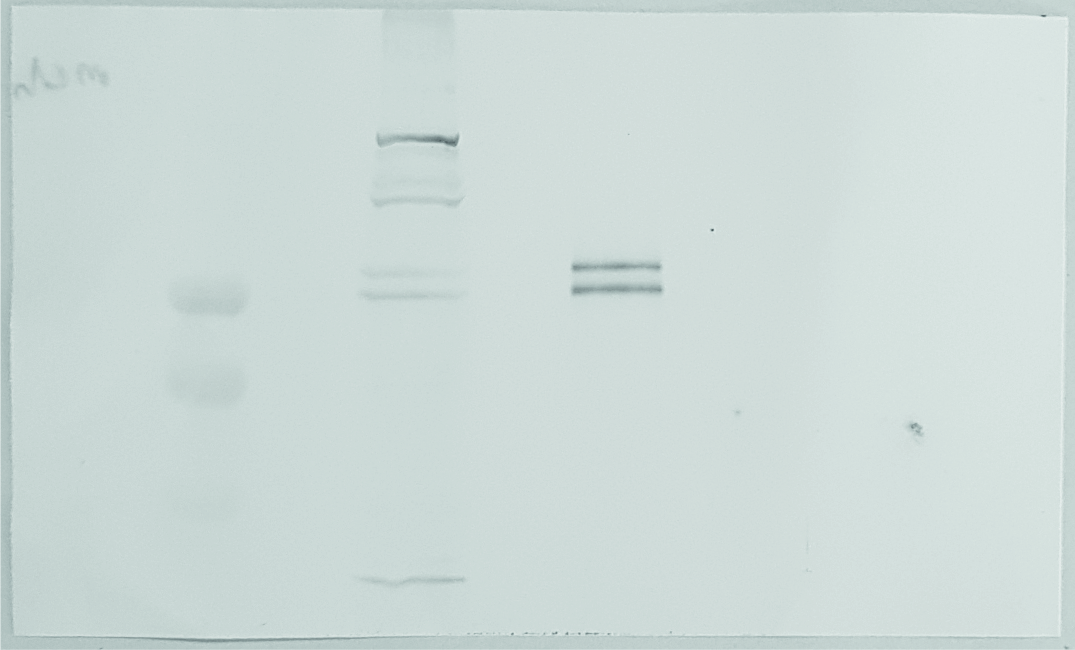

Supplement: Figure 7—source data 1. [file elife-89582-fig7-data1.zip › head co-IP anti-mCh.jpg]

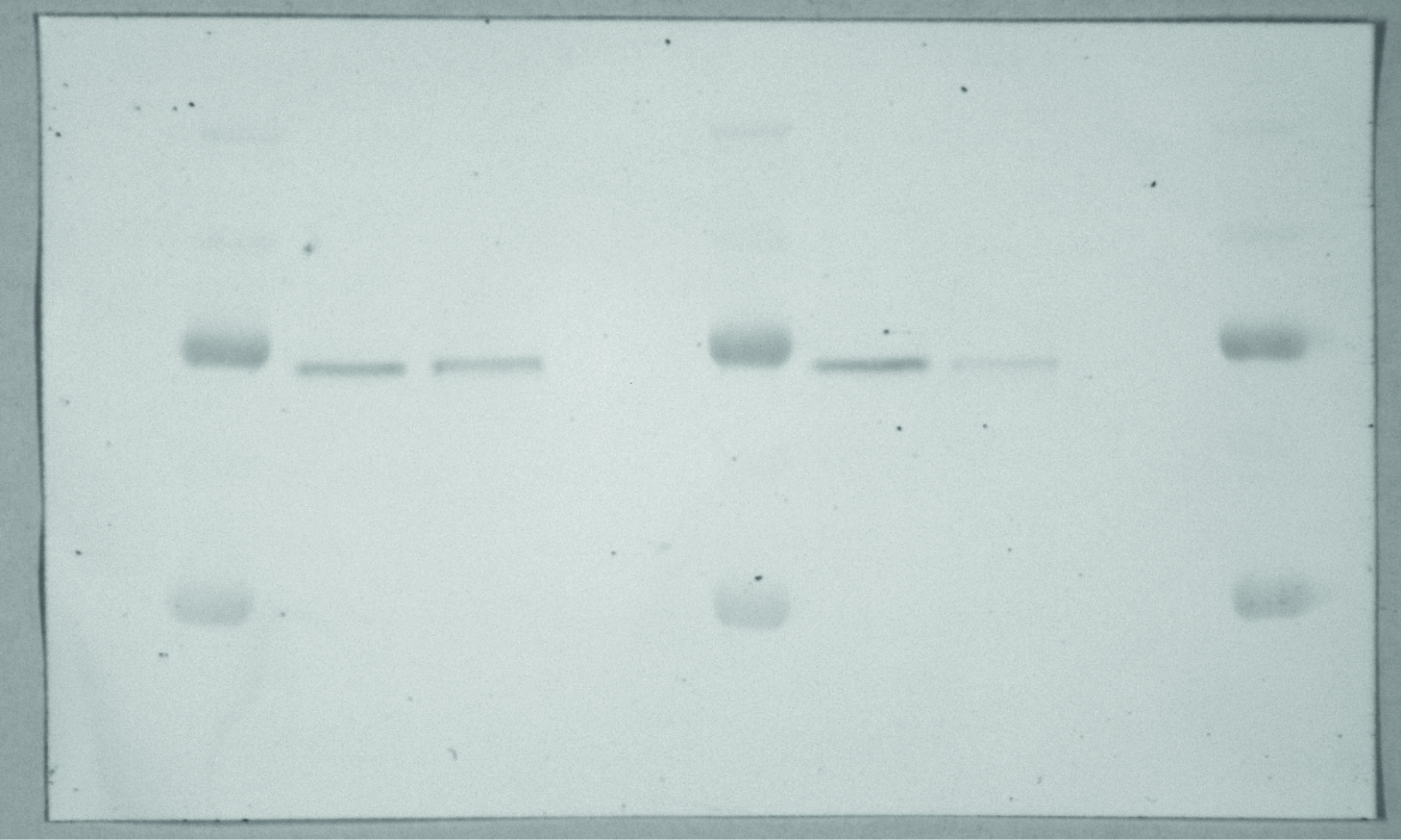

Supplement: Figure 7—source data 1. [file elife-89582-fig7-data1.zip › hemo co-IP anti-HA.jpg]

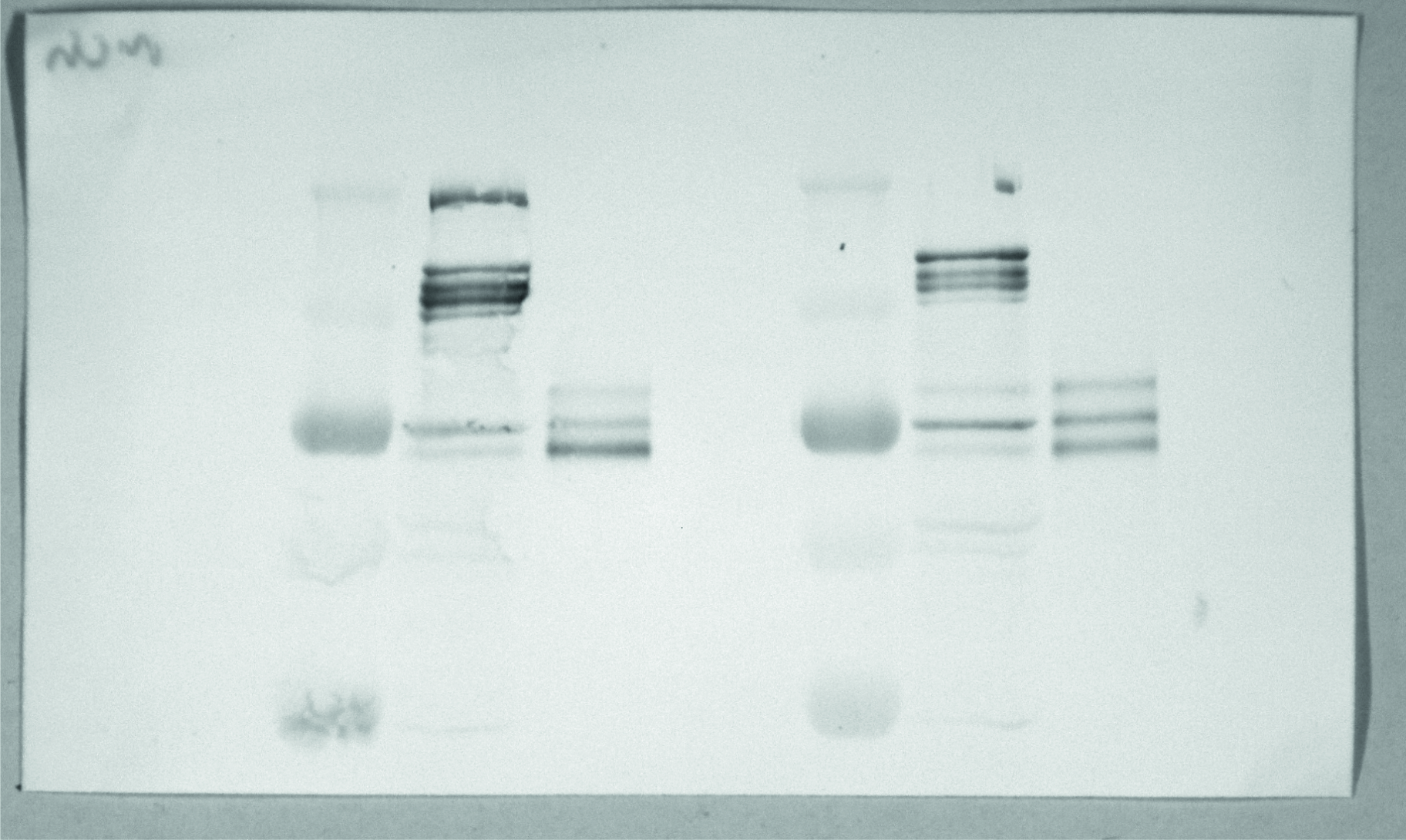

Supplement: Figure 7—source data 1. [file elife-89582-fig7-data1.zip › hemo co-IP anti-mCh.jpg]

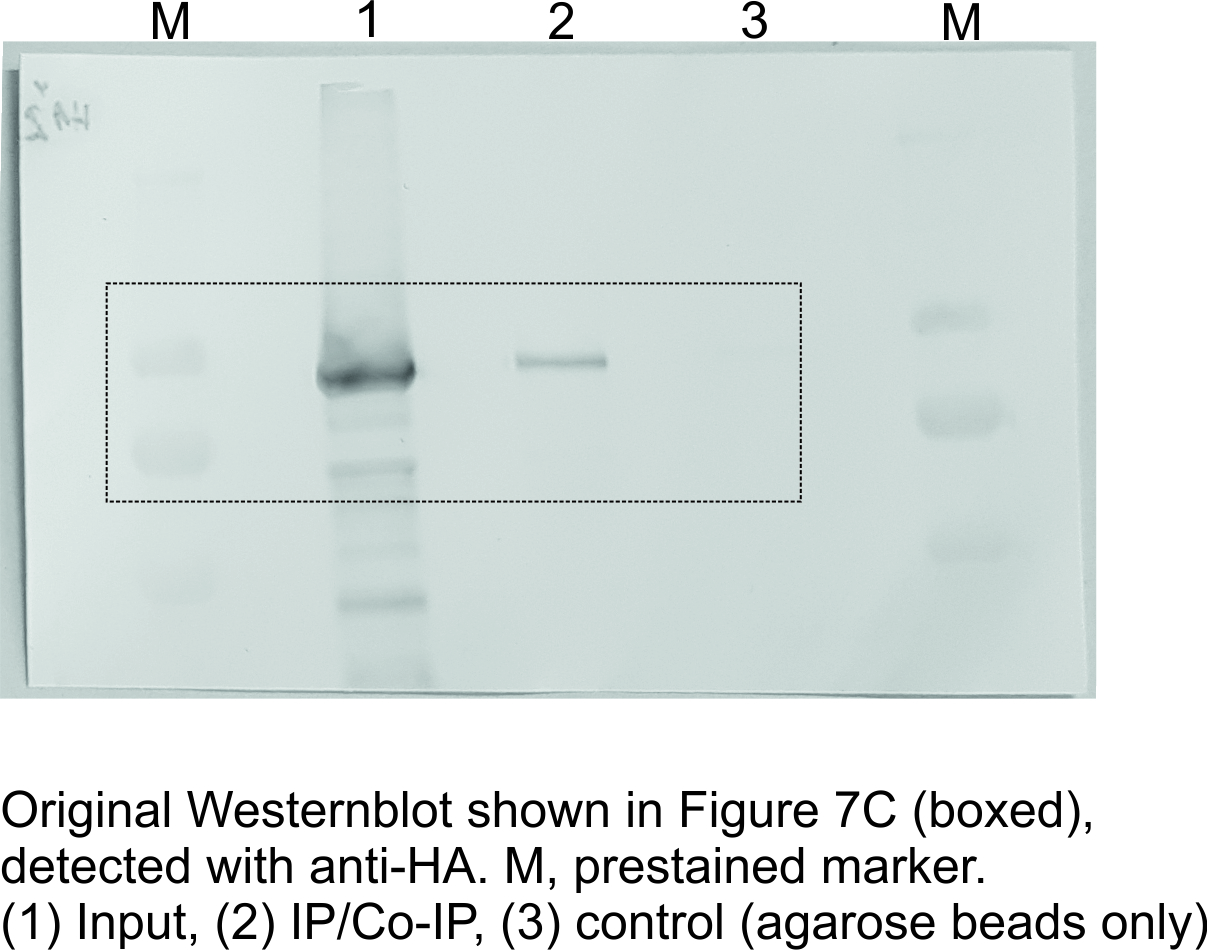

Supplement: Figure 7—source data 2. — Boxed areas correspond to the regions shown in the main figure. [file elife-89582-fig7-data2.zip › Figure 7C anti-HA.jpg]

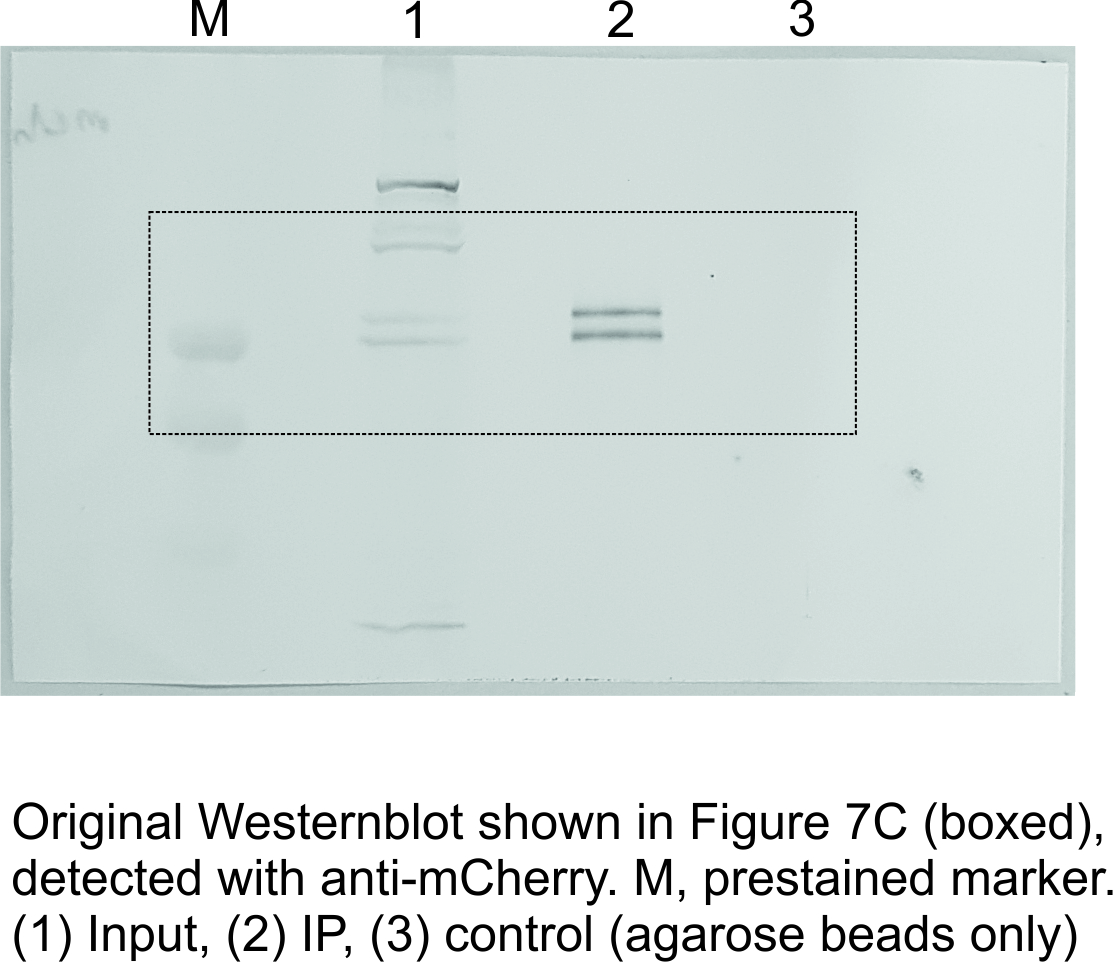

Supplement: Figure 7—source data 2. — Boxed areas correspond to the regions shown in the main figure. [file elife-89582-fig7-data2.zip › Figure 7C anti-mCh.jpg]

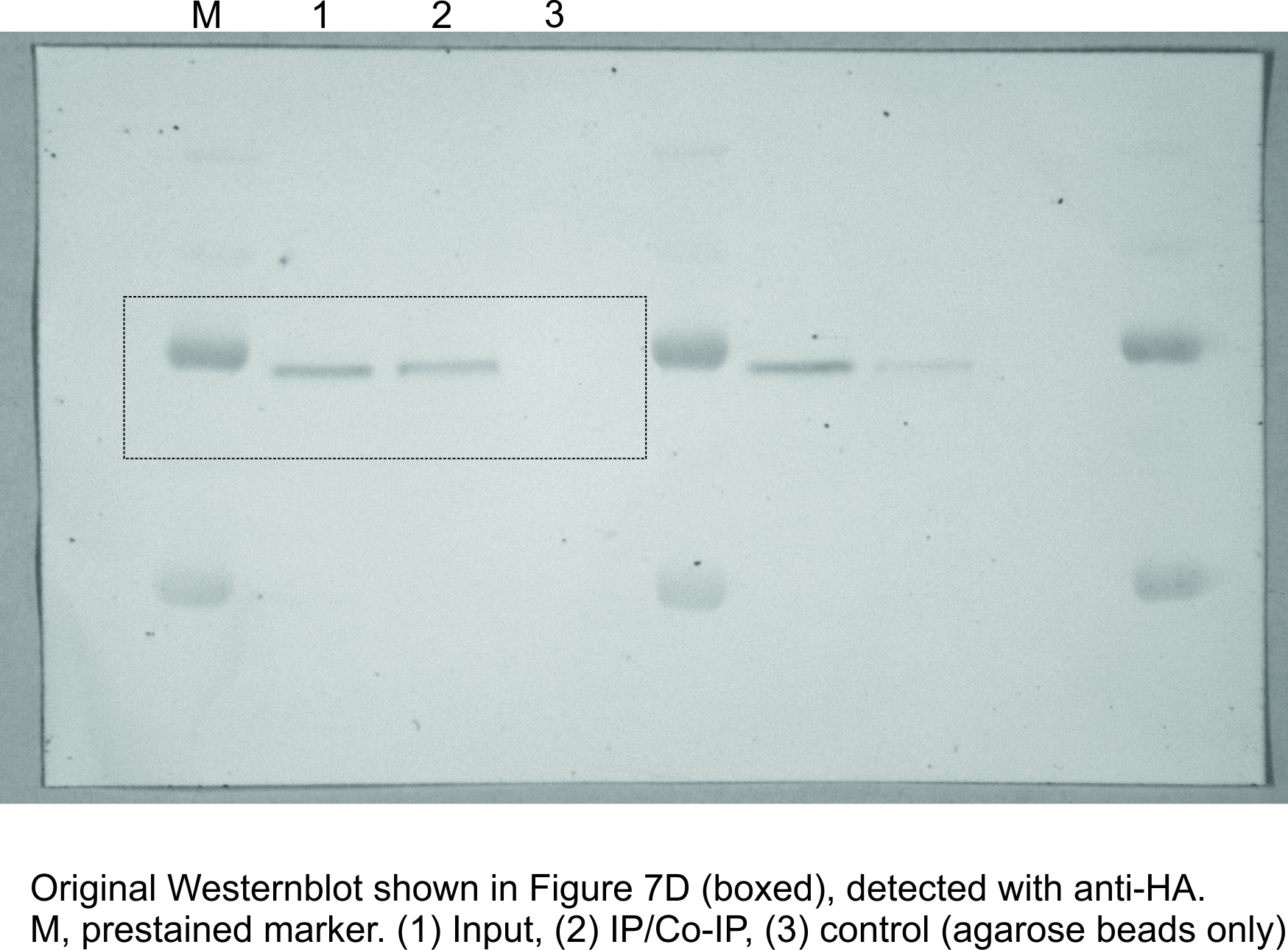

Supplement: Figure 7—source data 2. — Boxed areas correspond to the regions shown in the main figure. [file elife-89582-fig7-data2.zip › Figure 7D anti-HA.jpg]

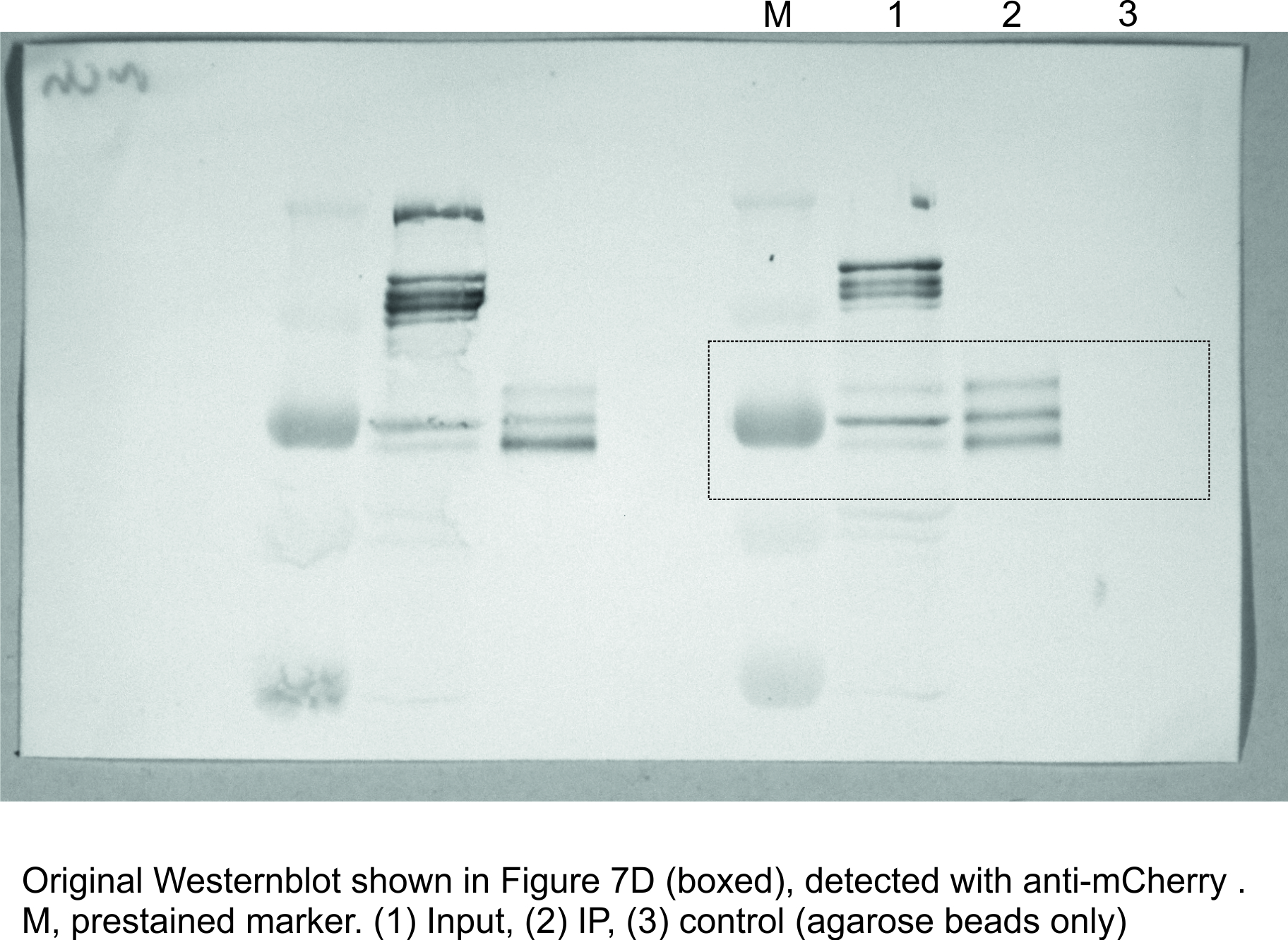

Supplement: Figure 7—source data 2. — Boxed areas correspond to the regions shown in the main figure. [file elife-89582-fig7-data2.zip › Figure 7D anti-mCh.jpg]

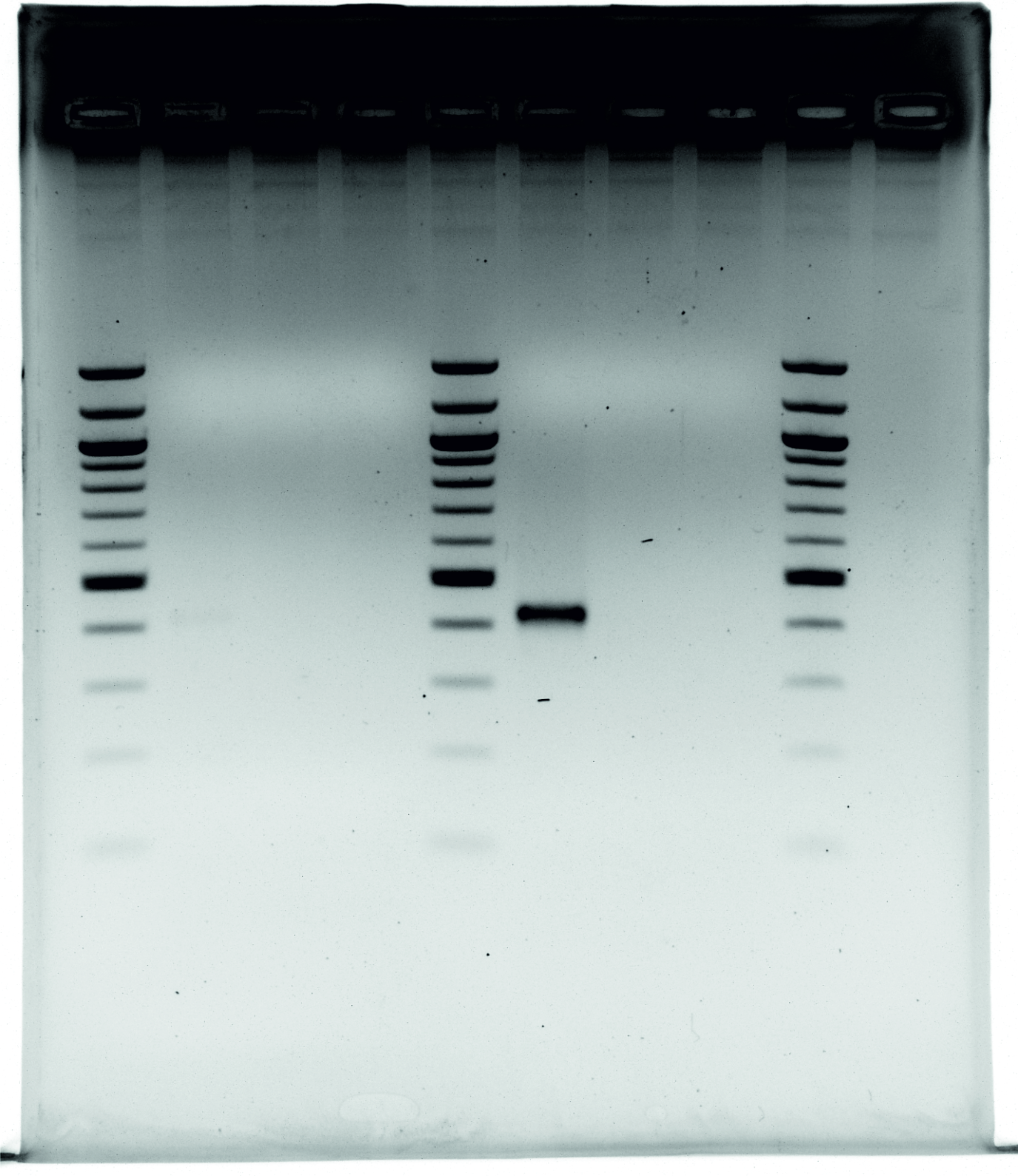

Supplement: Figure 8—figure supplement 1—source data 1. [file elife-89582-fig8-figsupp1-data1.zip › Figure 8-Figure Supplement 1-Source Data 1.jpg]

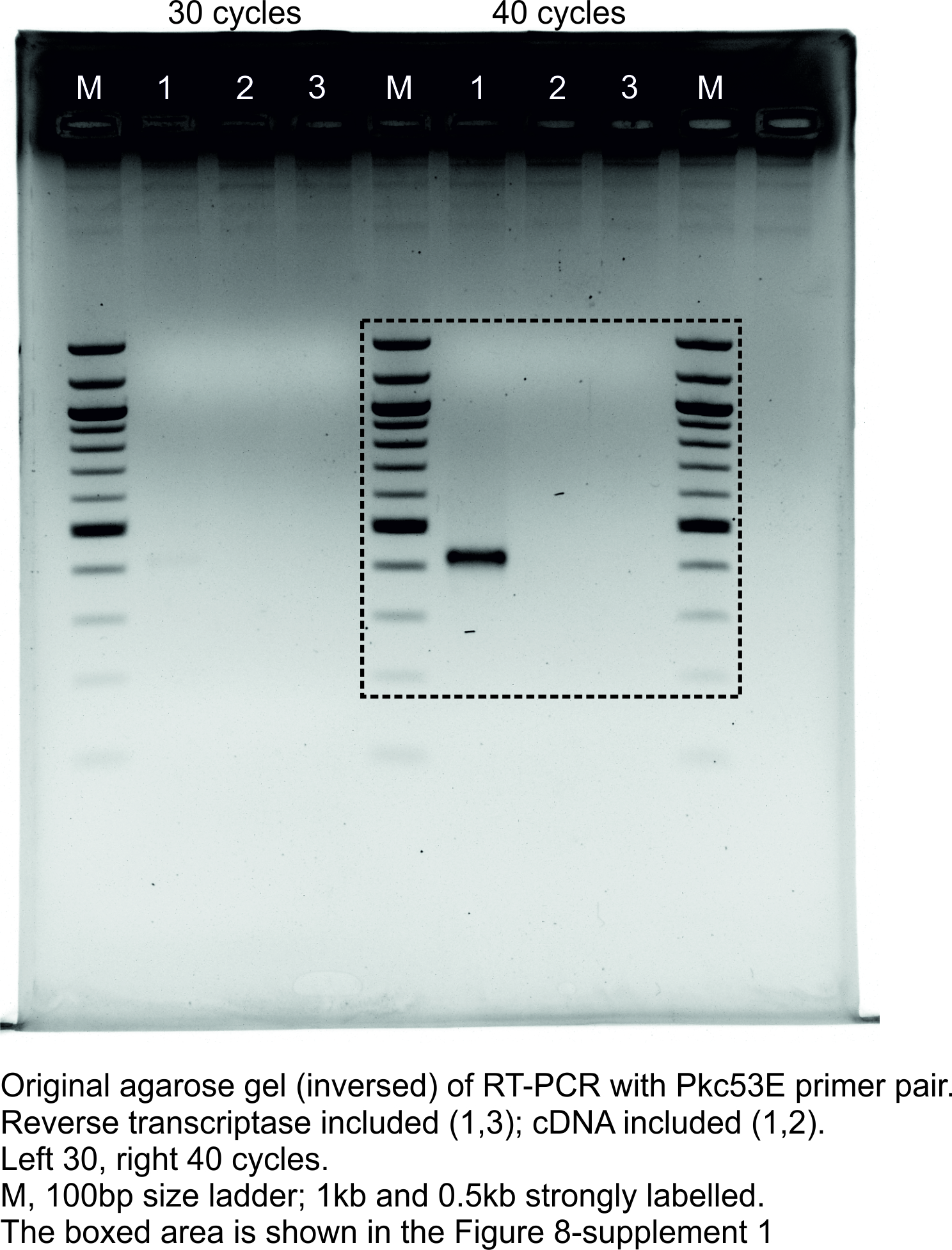

Supplement: Figure 8—figure supplement 1—source data 2. — Boxed area corresponds to region shown in the main figure. [file elife-89582-fig8-figsupp1-data2.zip › Figure 8-Figure Supplement 1-Source Data.jpg]
