# Supplementary material for: Inhibition of the Notch signal transducer CSL by Pkc53E-mediated phosphorylation to fend off parasitic immune challenge in Drosophila
Source: eLife. 2024 Nov 6;12:RP89582. doi: 10.7554/eLife.89582 (PMC11540305; doi:10.7554/eLife.89582)
Supplement: Supplementary file 1. — Computationally determined candidate Ser/Thr kinases predicted to pilot Serine 269 in Su(H). The list contains the human and the corresponding Drosophila candidates. [file elife-89582-supp1.docx]

**Supplementary file 1**

List of kinases predicted to recognize S269 in Su(H) as substrate *in silico*

| **Family of Kinase** | **Human** | ***Drosophila*** |
| --- | --- | --- |
|  | AKT1/AKT2 | Akt1 |
|  | GRK5 | Gprk2 |
|  | LATS1/LATS2 | Wts |
| **AGC** | PDK1 | Pdk1 |
|  | PRKCI | atypical PKC |
|  | PRKCD | Pkc delta |
|  | PRKG1 | For |
|  | RPS61K3/RSKp90 | S6kII |
|  | CAMK2A/CAMK2D | CamkII |
|  | MAPK3 | Par-1 |
| **CAMK** | MAPKAPK3 | Mapk-Ak2 |
|  | MYLK | Strn-Mlck |
|  | PASK | Pask |
|  | SIK2 | Sik2 |
|  | CDK9/CycK/  CDK9/CycT | Cdk9 |
| **CMGC** | GSK3-alpha | Sgg |
|  | GSK3-B | Gskt |
|  | MAP2K3 | Lic |
| **STE** | MST1/MST2 | Hpp |
|  | PAK4 | Mbt |
|  | AURA | AurA |
|  | CHK1 | Grp |
| **OPK** | DYRK1 | Mnb |
|  | DYRK2 | Dyrk3 |
|  | RAF1 | Raf |
|  | WNK | Wnk |
|  | ATM | Tefu |
|  | ATR | Mei-41 |
| **Protein kinase like** | HASPIN | Haspin |
|  | PRKB1 | Alc |

36 human candidate kinases, corresponding to 30 *Drosophila* kinases, were computationally predicted to have S269 in Su(H) as a target phospho-site using GPS 3.0 software.
