# Supplementary material for: Inhibition of the Notch signal transducer CSL by Pkc53E-mediated phosphorylation to fend off parasitic immune challenge in Drosophila
Source: eLife. 2024 Nov 6;12:RP89582. doi: 10.7554/eLife.89582 (PMC11540305; doi:10.7554/eLife.89582)
Supplement: Supplementary file 2. — List of Ser/Thr kinases that tested positive in accepting Drosophila Su(H) BTD as a substrate for phosphorylation in an in vitro assay. The list contains the 62 human and the corresponding 40 Drosophila candidates, highlighting the 10 from Drosophila also identified in the in silico screen. [file elife-89582-supp2.docx]

**Supplementary file 2**

List of kinases accepting the BTD domain of Su(H) as substrate *in vitro*

| **Family of Kinase** | **Human** | ***Drosophila*** |
| --- | --- | --- |
|  | **AKT1/AKT2/AKT3/SGK2** | **Akt1** |
| **AGC** | PKC alpha/PKC beta1/  PKC beta2/ PKC gamma | Pkc53E |
|  | **PKC delta** | **PKC delta** |
|  | PKC nu | Pkd |
|  | CAMK1D/CAMK4 | CamkI |
|  | **CAMK2A/CAMK2B/**  **CAMK2D/CAMK2G** | **CamkII** |
| **CAMK** | CHK2 | Lok |
|  | **MAPKAPK3** | **MAPK-Ak2** |
|  | **SIK1/SIK2** | **Sik2** |
| **CkI** | TTBK1 | Asator |
|  | CDK1/CycA | Cdk1 |
|  | CDK1/CycE  CDK2/CycE  CDK3/CycE  CDK2/CycA | Cdk2 |
| **CMGC** | CDK7/CycH | Cdk7 |
|  | CDK8/CycC | Cdk8 |
|  | CLK2 | Doa |
|  | **GSK3-alpha** | **Sgg** |
|  | **GSK3-B** | **Gskt** |
|  | JNK2 | Bsk |
|  | CDC7/ASK | CG5790 |
|  | MAP4K4 | Msn |
|  | MAP4K5 | Hppy |
| **STE** | MEK1 | Dsor1 |
|  | **MST1/MST2** | **Hpp** |
|  | NEK3/NEK9 | Niki |
|  | STK39 | Fray |
|  | TAOK2 | Tao |
|  | ACV-R2A | Put |
|  | BMPR1A | Tkv |
|  | **B-RAF/B-RAFVE** | **Raf** |
|  | BUB1B | BubR1 |
|  | CK2-alpha1/  CK2-alpha2 | CkII alpha |
| **OPK** | HIPK1/HIPK2 | Hipk |
|  | IRAK4 | Pll |
|  | LIMK1/LIMK2 | Limk1 |
|  | MAP3K1/MAP3K9 | Slpr |
|  | PBK | CG8173 |
|  | TLK2 | Tlk |
|  | TSSK1 | CG14305 |
|  | WEE1 | Wee1 |
|  | **WNK1/WNK2** | **Wnk** |

62 out of 245 human Ser/Thr kinases tested (equates to 25%) were identified to phosphorylate the BTD of Su(H) *in vitro*. These 62 human kinases correspond to 40 different kinases in *Drosophila*. 20 of the human kinases, corresponding to 10 *Drosophila* kinases were also predicted by the in silico analysis (bold). The KinaseFinder Service was conducted by ProQinase (Freiburg Germany).
