# Supplementary material for: Inhibition of the Notch signal transducer CSL by Pkc53E-mediated phosphorylation to fend off parasitic immune challenge in Drosophila
Source: eLife. 2024 Nov 6;12:RP89582. doi: 10.7554/eLife.89582 (PMC11540305; doi:10.7554/eLife.89582)
Supplement: Supplementary file 3. — This file contains a list of the mutant alleles and RNAi strains of the Drosophila Ser/Thr kinases screened for alterations in crystal cell numbers with identifier, reference and/or source (BL, Bloomington Drosophila Stock Center; VDRC, Vienna Drosophila Resource Center). [file elife-89582-supp3.docx]

**Supplementary file 3**

Fly strains used for the larval crystal cell screen

| **Kinase** | **Allele/RNAi/others** | **Source/Identity** |
| --- | --- | --- |
| Akt1 | *Akt^04226^*/TM6ubi-GFP | Balancer changed from BL11627 (RRID:BDSC_11627) |
| Alc | *alc^Ad2^*/CyO-GFP | Balancer changed from BL5510 (RRID:BDSC_5510) |
| Asator | TRIP_HMC04184attP2  dsRNA in Valium20 | BL55902 (RRID:BDSC_55902) |
| Bsk | TRIP_HMC03539attP2  dsRNA in Valium20 | BL53310 (RRID:BDSC_53310) |
| BubR1 | UAS-BubR1^DN^  C-terminal truncated form | BL8380 (RRID:BDSC_8380) |
|  | UAS-BubR1^DN^  C-terminal truncated form | BL8382 (RRID:BDSC_8380) |
| CamkI | TRIP_JF02268attP2  dsRNA in Valium10 | BL26726 (RRID:BDSC_26726) |
| CamkII | TRIP_GL00237attP2/TM6B  dsRNA in Valium22 | BL35330 (RRID:BDSC_26726) |
|  | UAS-CamKII^T287A^  Dominant negative version | BL29663 (RRID:BDSC_29663) |
| Cdk1 | *Cdk1^E1-23^*/CyO-GFP | Balancer changed from BL6629 (RRID:BDSC_6629) |
| Cdk2 | TRIP_HM05163attP2  dsRNA in Valium10 | BL28952 (RRID:BDSC_28952) |
| Cdk7 | *Cdk7^del^*/FM7-GFP | Balancer changed from BL4557 (RRID:BDSC_4557) |
| Cdk8 | TRIP_HMS05476attP40  dsRNA in Valium20 | BL67010 (RRID:BDSC_67010) |
| CG5790 | TRIP_HMJ23933attP40/CyO-GFP dsRNA in Valium20 | Balancer changed from BL62453 (RRID:BDSC_62453) |
| CG8173 | TRIP_JF01161attP2  dsRNA in Valium1 | BL31586 (RRID:BDSC_31586) |
| CG14305 | TRIP_HMC05158attP40  dsRNA in Valium20 | BL62151 (RRID:BDSC_62151) |
| CkII ⍺ | TRIP_JF01436attP2  dsRNA in Valium1 | BL31645 (RRID:BDSC_31645) |
| Doa | TRIP_HMC04193attP2  dsRNA in Valium20 | BL55908 (RRID:BDSC_55908) |
|  | GD8588 dsRNA in pUASt | VDRC_19066 |
| Drak | *Drak^del^* | Neubuesser and Hipfner, 2010 |
| Dsor1 | TRIP_HMS00710 attP2/TM6B  dsRNA in Valium20 | Balancer changed from BL32920 (RRID:BDSC_32920) |
|  | KK102276 dsRNA in pUASt | VDRC_107276 |
| Dyrk3 | TRIP_HMC04155attP2  dsRNA in Valium20 | BL55882 (RRID:BDSC_55882) |
| Fray | TRIP_HMS01794attP2  dsRNA in Valium20 | BL38327 (RRID:BDSC_38327) |
| Gskt | TRIP_HMC05795attP2  dsRNA in Valium20 | BL64922 (RRID:BDSC_64922) |
| Hipk | TRIP_HMC05078attP40  dsRNA in Valium20 | BL60084 (RRID:BDSC_60084) |
| Hpo | GD1570 dsRNA in pUASt | VDRC_7823 |
|  | KK101704 dsRNA in pUASt | VDRC_104169 |
| Lic | GD7546 dsRNA in pUASt | VDRC_20166 |
| MAPk-Ak2 | TRIP_HMS04456attP40  dsRNA in Valium20 | BL57013  (RRID:BDSC_57013) |
| Mnb | KK102642 dsRNA in pUASt | VDRC_107066 |
| Msn | KK108948 dsRNA in pUASt | VDRC_101517 |
|  | pValium20_attP40 shRNA | VDRC_330049 |
| Niki | TRIP_HMS01477attP2  dsRNA in Valium20 | BL35735 (RRID:BDSC_35735) |
| Par-1 | *par-1^k06323^*/CyO-GFP | Balancer changed from BL10615 (RRID:BDSC_10615) |
| Pdk1 | KK108363 dsRNA in pUASt | VDRC_109812 |
|  | TRIP_JF02807attP2  dsRNA in Valium10 | BL27725 (RRID:BDSC_27725) |
| Pkc53E | *Pkc53E^Δ28^* | BL80988 (RRID:BDSC_80988) |
|  | TRIP_JF02641 attP2  dsRNA in Valium10 | BL27491 (RRID:BDSC_27491) |
|  | TRIP_HMS01195 attP2  dsRNA in Valium20 | BL34716 (RRID:BDSC_34716) |
| Pkc98E | TRIP_JF02470 attP2  dsRNA in Valium10 | BL29311 (RRID:BDSC_29311) |
|  | TRIP_GL00174 attP2  dsRNA in Valium22 | BL35275 (RRID:BDSC_35275) |
| Pkd | *PKD^cl4^* | BL93864 (RRID:BDSC_93864) |
|  | TRIP_JF03144attP2  dsRNA in Valium10 | BL28717  (RRID:BDSC_28717) |
| Pll | KK102624 dsRNA in pUASt | VDRC_103774 |
| Put | TRIP_JF02664 attP2  dsRNA in Valium10 | BL27514 (RRID:BDSC_27491) |
| Raf | *raf^12^*/FM7-GFP | Balancer changed from BL5779 (RRID:BDSC_5779) |
| S6k | *S6k^I-1^*/TM6B | BL32552 (RRID:BDSC_32552) |
| Sgg | *Sgg^M1-1^/FM7-GFP* | Balancer changed from BL5402 (RRID:BDSC_5402) |
| Sik2 | TRIP_HMC04153 attP2  dsRNA in Valium20 | BL55880 (RRID:BDSC_55880) |
| Slpr | *slpr^3P5^/ FM7-GFP* | Balancer changed from BL58795 (RRID:BDSC_58795) |
|  | *slpr^BS06^/ FM7-GFP* | Balancer changed from BL58807 (RRID:BDSC_58807) |
|  | KK100726 dsRNA in pUASt | VDRC_106449 |
|  | GD9771/CyO-GFP  dsRNA in pUASt | VDRC_33518 |
| Tefu | TRIP_HMS02790 attP40  dsRNA in Valium20 | BL44073 (RRID:BDSC_44073) |
| Tkv | *tkv^1^* | BL427 (RRID:BDSC_427) |
| Wee | TRIP_HMC03331 attP40  dsRNA in Valium20 | BL51776 (RRID:BDSC_51776) |
| Wnk | TRIP_HMJ02087 attP40  dsRNA in Valium20 | BL42521 (RRID:BDSC_42521) |
| **Gal4 driver/control lines** | **Allele/RNAi/other information** | **Source/Identity** |
| *Hml*-Gal4 | Expresses Gal4 in lymph glands and circulating hemocytes | BL30141 (RRID:BDSC_30141) |
| *white-*RNAi | TRIP_JF01574attP2 dsRNA in Valium1 vector | BL31231 (RRID:BDSC_31231) |
