# Supplementary material for: Inhibition of the Notch signal transducer CSL by Pkc53E-mediated phosphorylation to fend off parasitic immune challenge in Drosophila
Source: eLife. 2024 Nov 6;12:RP89582. doi: 10.7554/eLife.89582 (PMC11540305; doi:10.7554/eLife.89582)
Supplement: Supplementary file 4. — This file contains the results from the larval crystal cell screen. The list displays the Ser/Thr kinases and the relevant controls tested in the screen, the alleles or RNAi settings used, the average crystal cell number of the tested mutant and the percentage gain or loss of crystal cells relative to the control, SD, and sample size. Heated larvae of kinase mutants and/or hml-Gal4::UAS-kinase-RNAi/UAS-kinaseDN genotypes were counted for the appearance of melanised crystal cells (cc) in the last two segments. If UAS-transgenes were used, the number of crystal cells was compared between uninduced (UAS-line alone) and induced with hml-Gal4. Mutant genotypes were related to the Su(H)gwt wild-type control. [file elife-89582-supp4.docx]

**Supplementary file 4**

Larval crystal cell screen

Heated larvae of kinase mutants and/or *hml*-Gal4::UAS-kinase-RNAi/UAS-kinase^DN^ genotypes were investigated for the appearance of melanized crystal cells (cc) in the last two segments. If UAS-transgenes were used, the number of crystal cells was compared between uninduced (UAS-line alone) and induced with *hml*-Gal4. Mutant genotypes were related to the *Su(H)^gwt^* wild-type control.

Evaluation of cc numbers was ranged according to the following categories:

(1) No cc present, or less cc in comparison to controls and reference lines (at least 20%)

(2) cc number within the range of *Su(H)^gwt^* and/or reference lines (± 20% of reference)

(3) cc number increased within the range of *Su(H)^S269A^* (> 20-50% to reference)

(4) cc number strongly increased above *Su(H)^S269A^* level (> 50% to reference)

| **Kinase (family)** | **Genotype** | **# Crystal cells** | **SD** | **Sample size (n)** |
| --- | --- | --- | --- | --- |
|  | | | | |
| **(1) Reduced numbers** | | | | |
| Cdk1 (CMGC) | *cdk1^E1-23^* | 0.12 | 0.33 | 25 |
| Shaggy (CMGC) | *sgg^M1-1^* | 1.76 | 1.39 | 25 |
|  |  |  |  |  |
| Cdk7 (CMGC) | *cdk7^del^* | 71.68 | 17.52 | 44 |
| Raf (OPK) | *raf^12^* | 65.03 | 36.85 | 60 |
|  | | | | |
| **(2) Match control** | | | | |
| Control | *Su(H)^gwt^* | 99.80 | 23.15 | 69 |
| Control | *hml-Gal4* | 110.32 (+11%) | 28.92 | 71 |
| Control | *white-RNAi* | 116.41 | 18.79 | 44 |
|  | *hml::white-RNAi* | 110.67 (-5%) | 13.62 | 45 |
| Asator (CkI) | *Asator-RNAi* | 101.97 | 26.79 | 61 |
|  | *hml::Asator-RNAi* | 106.86 (+5%) | 42.51 | 77 |
| CamKI (CAMK) | *CamkI-*RNAi | 84.96 | 35.42 | 51 |
|  | *hml*::*CamkI-*RNAi | 76.44 (-11%) | 40.1 | 71 |
| Cdk2 (CMGC) | *cdk2-RNAi* | 133.26 | 29.39 | 54 |
|  | *hml::cdk2-RNAi* | 123.11 (-8%) | 35.44 | 61 |
| CG5790 (STE) | *CG5790-RNAi* | 143.64 | 53.16 | 59 |
|  | *hml::CG5790-RNAi* | 128.69 (-11%) | 32.96 | 67 |
| Dsor1 (STE) | *Dsor1-RNAi* (A) | 191.72 | 43.56 | 53 |
|  | *hml::Dsor1-RNAi* | 159.04 (-17%) | 42.65 | 57 |
|  | *Dsor1-RNAi* (B) | 151.86 | 34.92 | 56 |
|  | *hml::Dsor1-RNAi* | 146.19 (- 4%) | 32.72 | 52 |
| Fray (STE) | *fray-RNAi* | 124.45 | 31.30 | 51 |
|  | *hml::fray-RNAi* | 137.38 (+10%) | 40.13 | 65 |
| Hipk (OPK) | *hipk-RNAi* | 102.26 | 37.52 | 53 |
|  | *hml::hipk-RNAi* | 121.35 (+19%) | 42.54 | 74 |
| Hpo (STE) | *hpo-RNAi* (A) | 166.13 | 44.05 | 48 |
|  | *hml::hpo-RNAi* | 159.64 (- 4%) | 63.81 | 61 |
|  | *hpo-RNAi* (B) | 74.91 | 25.85 | 55 |
|  | *hml::hpo-RNAi* | 87.21 (+16%) | 42.58 | 62 |
| Lic (STE) | *lic-RNAi* | 80.53 | 29.37 | 45 |
|  | *hml::lic-RNAi* | 81.15 | 27.12 | 52 |
| MAPk-Ak2 (CAMK) | *MAPk-Ak2-RNAi* | 104.09 | 25.96 | 56 |
|  | *hml::MAPk-Ak2-RNAi* | 117.98 (+13%) | 31.27 | 52 |
| Mnb (OPK) | *mnb-RNAi* | 116.10 | 42.25 | 40 |
|  | *hml::mnb-RNAi* | 115.40 | 30.82 | 43 |
| Sik2 (CAMK) | *Sik2-RNAi* | 142.38 | 28.81 | 47 |
|  | *hml::Sik2-RNAi* | 151.47 (+6%) | 34.80 | 53 |
| Tefu (Protein kinase like) | *tefu-RNAi* | 56.69 | 18.12 | 55 |
|  | *hml*::*tefu-*RNAi | 56.41 | 28.15 | 61 |
| Wee1 (OPK) | *Wee1-RNAi* | 105.44 | 29.4 | 55 |
|  | *hml::Wee1-RNAi* | 121.37 (+15%) | 36.43 | 68 |
|  | | | | |
| **(3) Match *Su(H)^S269A^*** | | | | |
| Reference | *Su(H)^S269A^* | 135.77 (+36%) | 25.99 | 70 |
| Akt (AGC) | *akt1^04226^* | 135.1 (+36%) | 43.14 | 48 |
| BubR1 (OPK) | *BubR1^DN^* (A) | 77.92 | 20.09 | 52 |
|  | *hml::BubR1^DN^* | 105.14 (+35%) | 22.25 | 58 |
|  | *BubR1^DN^* (B) | 84.78 | 25.43 | 49 |
|  | *hml::BubR1^DN^* | 112.85 (+33%) | 34.11 | 48 |
| Bsk (CMGC) | *bsk-RNAi* | 110.23 | 41.19 | 62 |
|  | *hml::bsk-RNAi* | 149.03 (+35%) | 35.84 | 71 |
| CamkII (CAMK) | *CamkII^DN^* | 163.97 | 30.53 | 58 |
|  | *hml:: CamkII^DN^* | 221.35 (+35%) | 54.03 | 46 |
|  | *CamkII-RNAi* | 152.96 | 49.05 | 50 |
|  | *hml:: CamkII-RNAi* | 188.85 (+23%) | 61.87 | 68 |
| Cdk8 (CMGC) | *cdk8-RNAi* | 115.63 | 29.36 | 57 |
|  | *hml::cdk8-RNAi* | 170.63 (+47%) | 31.85 | 56 |
| CG14305 (OPK) | *CG14305-RNAi* | 96.49 | 27.27 | 43 |
|  | *hml::CG14305-RNAi* | 134.55 (+39%) | 36.37 | 65 |
| Doa (CMGC) | *Doa-RNAi (A)* | 143.35 | 22.56 | 63 |
|  | *hml::Doa-RNAi* | 174.02 (+21%) | 36.58 | 60 |
|  | *Doa-RNAi (B)* | 114.20 | 23.34 | 46 |
|  | *hml::Doa-RNAi* | 170.42 (+49%) | 35.73 | 45 |
| Dyrk3 (OPK) | *Dyrk3-RNAi* | 105.07 | 27.65 | 60 |
|  | *hml::Dyrk3-RNAi* | 151.95 (+45%) | 35.43 | 59 |
| Gskt (CMGC) | *gskt-RNAi* | 98.60 | 35.01 | 50 |
|  | *hml::gskt-RNAi* | 137.46 (+39%) | 33.41 | 63 |
| Msn (STE) | *msn-RNAi* (A) | 82.83 | 36.11 | 53 |
|  | *hml::msn-RNAi* | 122.58 (+48%) | 47.36 | 53 |
|  | *msn-RNAi* (B) | 102.79 | 26.29 | 63 |
|  | *hml::msn-RNAi* | 135.92 (+32%) | 34.99 | 62 |
| Niki (STE) | *niki-RNAi* | 120.87 | 29.91 | 47 |
|  | *hml::niki-RNAi* | 147.0 (+22%) | 53.59 | 51 |
| Pdk1 (AGC) | *Pdk1-RNAi* (A) | 130.33 | 30.34 | 48 |
|  | *hml::Pdk1-RNAi* | 188.52 (+45%) | 33.82 | 50 |
|  | *Pdk1-RNAi* (B) | 123.83 | 30.06 | 46 |
|  | *hml:: Pdk1-RNAi* | 168.24 (+47%) | 28.89 | 46 |
| Pkc53E (AGC) | *Pkc53E^△28^* | 137.40 (+38%) | 47.92 | 70 |
|  | *PKc53E-RNAi* (A) | 140.42 | 25,75 | 55 |
|  | *hml::Pkc53E-RNAi* | 185.09 (+32%) | 34.26 | 70 |
|  | *PKc53E-RNAi* (B) | 111.21 | 33.79 | 52 |
|  | *hml::Pkc53E-RNAi* | 163.29 (+47%) | 34.59 | 70 |
| Pkc98E (AGC) | *Pkc98E-RNAi* (A) | 81.03 | 35.02 | 60 |
|  | *hml::Pkc98E-RNAi* (A) | 111.52 (+38%) | 40.73 | 54 |
|  | *Pkc98E-RNAi* (B) | 126.25 | 30.84 | 61 |
|  | *hml::Pkc98E-RNAi* (B) | 165.67(+31%) | 29.49 | 85 |
| PKD (AGC) | *PKD^cl4^* | 137.23 (+38%) | 31.59 | 60 |
|  | *PKD-RNAi* | 125.85 | 30.45 | 47 |
|  | *hml::PKD-RNAi* | 161.18 (+28%) | 48.40 | 60 |
| Pll (TKL) | *pll-RNAi* | 125.76 | 41.70 | 55 |
|  | *hml::pll-RNAi* | 162.76 (+29%) | 42.52 | 55 |
| Put (Protein kinase like) | *put-RNAi* | 129.04 | 28.03 | 50 |
|  | *hml::put-RNAi* | 176.02 (+36%) | 41.26 | 45 |
| S6K (AGC) | *S6k^I-1^*/+ (no hz) | 145.1 (+45%) | 36.07 | 58 |
| Wnk (OPK) | *Wnk-RNAi* | 89.89 | 26.25 | 70 |
|  | *hml::Wnk-RNAi* | 127.10 (+41%) | 29.81 | 70 |
|  | | | | |
| **(4) Highly increased numbers** | | | | |
| Alc (Protein kinase like) | *alc^Ad2^*/+ (no hz) | 158.75 (+59%) | 54.65 | 52 |
| CG8173 (OPK) | *CG8173-RNAi* | 57.98 | 17.70 | 58 |
|  | *hml::CG8173-RNAi* | 114.85 (+98%) | 26.13 | 53 |
| CkII⍺ (OPK) | *CkII⍺-RNAi* | 86.43 | 27.18 | 63 |
|  | *hml::CkII⍺-RNAi* | 169.7 (+96%) | 34.86 | 63 |
| Drak (CAMK) | *drak^del^* | 151.62 (+52%) | 39.43 | 61 |
| Par-1 (CAMK) | *par-1^K06323^*/+ (no hz) | 151.6 (+52%) | 48.49 | 58 |
| Slpr (STE) | *slpr^B506^/slpr^3P5^* | 155.08 (+56%) | 36.12 | 50 |
|  | *slpr-RNAi* (A) | 49.64 | 16.91 | 47 |
|  | *hml:: slpr-RNAi* | 101.32 (+104%) | 29.55 | 50 |
|  | *slpr-RNAi* (B) | 106.69 | 25.09 | 59 |
|  | *hml:: slpr-RNAi* | 170.76 (+60%) | 26.57 | 59 |
| Tkv (OPK) | *tkv^1^* | 173.46 (+74%) | 43.04 | 54 |
