# Supplementary material for: Inhibition of the Notch signal transducer CSL by Pkc53E-mediated phosphorylation to fend off parasitic immune challenge in Drosophila
Source: eLife. 2024 Nov 6;12:RP89582. doi: 10.7554/eLife.89582 (PMC11540305; doi:10.7554/eLife.89582)
Supplement: Supplementary file 5. — This file contains a list of oligonucleotides used for cloning, mutagenesis, and verification of constructs, as well as for RT-PCR and qRT-PCR analyses, including PCR conditions. [file elife-89582-supp5.docx]

**Supplementary file 5**

List of Oligonucleotides

| **Primer** | **Sequence 5‘ -> 3‘** | **Purpose** | **PCR conditions** |
| --- | --- | --- | --- |
| ***Su(H) cloning and verification*** | | | |
| Su(H) BTD_UP | AAG GGA TCC TCG CTA AAG AAT GCC GAT CTG TG | BTD-amplification with 5’ *Bam*HI and 3’ *Eco*RI sites for subcloning into pGEX-2T | 52.5°C Annealing;  Product length:  525 bp |
| Su(H) BTD_LP | CAT GAA TTC TCA GAA CTG GTA CTC AGC CTT GTC GG |  |  |
| Myc-Tag_UP | AAT TCA TGG AGC AGA AGC TGA TCT CGG AGG AGG ATC TAG AGC AGA AGC TGA TCT CGG AGG AGG ATC TAG AGC | | Addition of myc-tag to Su(H) cDNA by insertion of annealed primers via *Eco*RI |
| Myc-Tag_LP | AAT TGC TCT AGA TCC TCC TCC GAG ATC AGC TTC TGC TCT AGA TCC TCC TCC GAG ATC AGC TTC TGC TCC ATG | |  |
| attR UP | CGG GGG ATC CAC TAG TTC TAG ATG TAG G | Verification of *Su(H)* genome engineered flies | 57°C Annealing;  Product length:  851 bp |
| Su(H) attR LP | CGC GCA TAG TTG TGC TCC CTG TTC G |  |  |
| 5‘Exon4 | TTA TGC CAC CGG TCT GAC CTT CA | Verification of *Su(H)*-mCherry tag | 60°C Annealing;  Product length:  1344 bp |
| Su(H) flox  LP | CAA CCG CAT CTA AAA ATC GTC TAT AAA CTT ACA TC |  |  |
| 5’Exon2 | CCC AAA AGT CCT ATG GCA ATG A | Differentiation of *Su(H)^gwt^* versus *Su(H)^S269A^* via *Alw44*I digest | 59°C Annealing;  Product length:  1.5kb in wild type vs 1.1+0.41 in mutant |
| Su(H) hom-reg LP | GGA TAA GCC GCT ACC ATG ACT ATT |  |  |
| ***Pkc53E* cloning, verification and mutagenesis** | | | |
| 3HA-Tag_UP  *Acc65*I/*Xho*I | GTA CCA TGT ATC CCT ATG ATG TGC CAG ACT ATG CTG GCT ATC CAT ATG ATG TTC CTG ATT ATG CTG GAT ACC CTT ATG ATG TGC CAG ACT ATG CCC | | Generation of pBT -3HA by insertion of annealed primers into Acc65I/XhoI of pBT |
| 3HA-Tag_LP  *Acc65*I/*Xho*I | TCG AGG GCA TAG TCT GGC ACA TCA TAA GGG TAT CCA GCA TAA TCA GGA ACA TCA TAT GGA TAG CCA GCA TAG TCT GGC ACA TCA TAG GGA TA**C** ATG | |  |
| *Xho*I_Pkc53E_UP | TTT ACT CGA GAT GTC GGA GGG CAG | Amplification of Pkc53E cDNA with 5’ *Xho*I and 3’ *Xba*I sites for subcloning into pBT-3HA vector | 59°C Annealing;  Product length:  2.1 kb |
| *Xba*I_Pkc53E_LP | CTG ATC TAG ACT ATG GGC TGA AAA CAT ATT CG |  |  |
| *Pkc53E Exon1_UP* | CGG AGG GCA GCG ATA ACA ACG G | Identification of Pkc53E null mutant | 59°C Annealing;  Product length:  553 kb in wild type, none in mutant |
| *Pkc53E Exon1_LP* | CAA GTG CCG TGG AGG GAA GTG GG |  |  |
| *Pkc53E_A34E_UP* | CCG CAA AGG **AGA G**CT CAA GAA GAA G | Site directed mutagenesis *A34E* | 61°C Annealing |
| *Pkc53E_A34E_LP* | AGG CGG GAC TTC ATT TTG |  |  |
| *Pkc53E_T508D_UP* | CGG TAC CCC TGA TTA CAT TGC TCC AG | Site directed mutagenesis *T508E* | 60°C Annealing |
| *Pkc53E_ T508D_LP* | CAG AA**A TC**C TTT GTG GTC TTA TCA CC |  |  |
| *Pkc53E_T650E_UP* | **GAT** CCC ACG GAC AAG GTG TTT ATG | Site directed mutagenesis *T650E* | 58°C Annealing |
| *Pkc53E_ T650E_LP* | CAG ATC TGT TTT CTC TGA TGT GAA CTG |  |  |
| *Pkc53E_S669D_UP* | AAT CCC GAG TAT GTT TTC AGC CCA TAG TC | Site directed mutagenesis *S669D* | 61°C Annealing |
| *Pkc53E_ S669D_LP* | CAT GTA **GTC** GAA GCC AAC GAA TTC CGA C |  |  |
| ***Pkc53E RT-PCR and qRT-PCR*** | | | |
| Pkc53E_RT-PCR UP | CTG CGG TAC ACC TGA TTA CAT TGC TC | RT-PCR for Pkc53E transcript | 58°C Annealing;  Product length:  430 bp |
| Pkc53E_RT-PCR LP | CGT GGG CGT CAA GTC TGT TTT CT |  |  |
| Tub56D_229 UP | GAA CCT ACC ACG GTG ACA GCG A | RT-PCR for *Tubulin56D* transcript | 65°C Annealing;  Product length:  299 bp |
| Tub56D_507 LP | GAA GCC AAG CAG GCA GTC GCA |  |  |
| GFP_1 UP | GCC ACA AGT TCA GCG TGT CCG | qRT-PCR for NRE-GFP transcript |  |
| GFP_1 Rev | GTA GGT CAG GGT GGT CAC GAG GG |  |  |
| atilla_4 UP | AAA CAA GTG ATT TTC GTG CTC CT | qRT-PCR for *atilla* transcript | PP7306 |
| atilla_4 Rev | CGC GGA TGT TAG AGG CAG A |  |  |
| cyp33_2 UP | CTC TGC GGA CGC ACA ATT C | qRT-PCR for *cyp33* transcript | PP14577 |
| cyp33_2 Rev | TGC AAC CAG TCG TCA TCT GC |  |  |
| Tbp_1 UP | TAA GCC CCA ACT TCT CGA TTC C | qRT-PCR for *Tbp* transcript | PP1556 |
| Tbp_1 Rev | GCC AAA GAG ACC TGA TCC CC |  |  |
| ***genotyping/verification*** | | | |
| Gal4 UP | CCG TCA CAG ATA GAT TGG CTT | Presence of Gal4 driver | 55°C Annealing;  Product length:  677 bp |
| Gal4 LP | AAG ATG TAG GGC TGT CAC CAA |  |  |
